# Ambient PM_2.5_ and risk of emergency room visits for myocardial infarction: impact of regional PM_2.5_ oxidative potential: a case-crossover study

**DOI:** 10.1186/s12940-016-0129-9

**Published:** 2016-03-24

**Authors:** Scott Weichenthal, Eric Lavigne, Greg Evans, Krystal Pollitt, Rick T. Burnett

**Affiliations:** Health Canada, 269 Laurier Ave West, Ottawa, K1A 0K9 Ontario Canada; University of Toronto, 200 College St, Toronto, M5S 3E5 Ontario Canada; University of Massachusetts, 686 North Pleasant Street, Amherst, Massachusetts 01003 USA

**Keywords:** Particulate matter, Oxidative potential, Myocardial infarction, Case-crossover

## Abstract

**Background:**

Regional differences in the oxidative potential of fine particulate air pollution (PM_2.5_) may modify its impact on the risk of myocardial infarction.

**Methods:**

A case-crossover study was conducted in 16 cities in Ontario, Canada to evaluate the impact of regional PM_2.5_ oxidative potential on the relationship between PM_2.5_ and emergency room visits for myocardial infarction. Daily air pollution and meteorological data were collected between 2004 and 2011 from provincial monitoring sites and regional estimates of glutathione (OP^GSH^) and ascorbate-related (OP^AA^) oxidative potential were determined using an acellular assay based on a synthetic respiratory tract lining fluid. Exposure variables for the combined oxidant capacity of NO_2_ and O_3_ were also examined using their sum (O_x_) and a weighted average (O_x_^wt^) based on their redox potentials.

**Results:**

In total, 30,101 cases of myocardial infarction were included in the analysis. For regions above the 90^th^ percentile of OP^GSH^ each 5 μg/m^3^ increase in same-day PM_2.5_ was associated with a 7.9 % (95 % CI: 4.1, 12) increased risk of myocardial infarction whereas a 4.1 % (95 % CI: 0.26, 8.0) increase was observed in regions above the 75^th^ percentile and no association was observed below the 50^th^ percentile (*p*-interaction = 0.026). A significant 3-way interaction was detected with the strongest associations between PM_2.5_ and myocardial infarction occurring in areas with high regional OP^GSH^ and high O_x_^wt^ (*p*-interaction < 0.001).

**Conclusions:**

Regional PM_2.5_ oxidative potential may modify the impact of PM_2.5_ on the risk of myocardial infarction. The combined oxidant capacity of NO_2_ and O_3_ may magnify this effect.

**Electronic supplementary material:**

The online version of this article (doi:10.1186/s12940-016-0129-9) contains supplementary material, which is available to authorized users.

## Background

Ambient fine particulate air pollution (PM_2.5_) contributes to acute cardiovascular morbidity including myocardial infarction [1–5]. While oxidative stress is known to play an important role in the cardiovascular health impacts of ambient PM_2.5_ [6–8], little is known about how regional differences in oxidative potential may modify the acute health effects of this pollutant. Moreover, few (if any) studies have examined the acute cardiovascular health impacts of the combined oxidant capacity of nitrogen dioxide (NO_2_) and ozone (O_3_) although recent evidence suggests that this measure may be advantageous to either pollutant on its own [9]. Therefore, given the ubiquitous nature of these three pollutants and the importance of oxidative stress pathways, it may be important to consider these parameters together in order to gain a more thorough understanding of the acute health impacts of PM_2.5_, particularly at low concentrations. Glutathione and ascorbate are important antioxidants in the body and act as a first line of defense against inhaled pollutants [8]. Moreover, several panel studies suggest that polymorphisms in glutathione s-transferase (GST) genes that reduce anti-oxidant capacity may increase susceptibility to the acute cardiovascular health effects of ambient PM_2.5_ [10–12]. As the oxidative properties of particulate air pollution vary both between [13] and within regions [14–17], such differences may translate into spatial differences in overall health effects. In particular, some evidence suggests that regional differences in PM_2.5_ oxidative potential may be explained in part by differences in particle composition as a European study of 19 cities reported strong correlations between the copper, iron, and zinc content of PM_2.5_ and ascorbate-related oxidative potential (OP^AA^) whereas aluminium and copper were associated with glutathione-related oxidative potential (OP^GSH^) [13]. However, other compounds such as polycyclic aromatic hydrocarbons and quinones likely also contribute regional differences in PM_2.5_ oxidative potential [7, 14, 18]. In this study, we examined the impact of regional differences in PM_2.5_ oxidative potential on the relationship between PM_2.5_ and the risk of emergency room visits for myocardial infarction. We also examined the relationship between the combined oxidant capacity (O_x_) of NO_2_ and O_3_ and the risk of myocardial infarction as well potential interactions between PM_2.5_, regional oxidative potential, and O_x_. To our knowledge this is the first study to examine how regional differences in oxidative potential may modify the acute health effects of PM_2.5_.

## Methods

### Study design

A time-stratified case-crossover study design [19] was used to estimate the impact of ambient PM_2.5_ on the risk of emergency room visits for myocardial infarction (ICD-10^th^ revision, Code I21-ST elevation(STEMI) and non-ST elevation (NSTEMI) myocardial infarction) in 16 cities in Ontario, Canada (Additional file 1: Table S1). Cases occurring between April 1, 2004 and December 31, 2011 were extracted from the National Ambulatory Care Reporting System (NACRS) database maintained by the Canadian Institute for Health Information (CIHI) along with demographic information including age and sex. All myocardial infarction cases with three digit postal codes corresponding to residences in these cities at the time of admission were eligible to be included in the analyses. Three-digit postal codes are specific to city boundaries (i.e. they do not overlap between cities). The NACRS database is estimated to capture more than 97 % of the emergency department visits in Ontario [20]. Ethics approval for this study was granted through a data sharing agreement between Health Canada and CIHI; patient consent was not necessary for ethics approval as only anonymized data were available to researchers in accordance with the agreement between Health Canada and CIHI.

### Daily Air pollution data

Daily average concentrations (April 1, 2004 to December 31, 2011) of ambient PM_2.5_, NO_2_ and O_3_ were collected in each city from fixed-site monitoring stations operated by the National Air Pollution Surveillance (NAPS) network and maintained by Environment Canada (i.e. PM_2.5_, NO_2_, and O_3_ data were collected from the same sites). If oxidative potential data were available for multiple monitors in a single city, daily average measurements of ambient air pollutants were extracted based on these single sites. Case and control periods in these cities were assigned exposures based on the monitoring station closest to the population-weighted centroid of each subject’s 3-digit postal code. Daily mean temperature and relative humidity data were also provided by Environment Canada using the average of measurements from all weather stations in each city. The combined oxidant capacity (O_x_) of NO_2_ and O_3_ was calculated as the sum of these pollutants (NO_2_ + O_3_) [9]. Redox-weighted oxidant capacity (O_x_^wt^) was calculated as a weighted average using redox potentials [21] as the weights (i.e. O_x_^wt^ = [(1.07 volts (V) × NO_2_) + (2.075 V × O_3_)]/3.145 V). The redox-weighted measure accounts for the fact that O_3_ is a stronger oxidant than NO_2_.

### City-level estimates of long-term PM_2.5_ oxidative potential

Integrated (i.e. averaged over a number of days/weeks) PM_2.5_ samples were collected between 2012 and 2013 from a total of 20 provincial monitoring sites located in the 16 cities included in this investigation. Only those sites with co-located daily air pollution and meteorological data were included in this analysis. The province of Ontario uses Thermo TEOM 1400AB monitors with Sample Equilibration Systems across its network at an operating temperature of 30 °C; therefore, only non-volatile chemical species are expected to be retained on the filters. However, previous assessments of non-volatile PM components have shown stable results for oxidative potential [17]. City-level estimates of PM_2.5_ oxidative potential were based on a mean duration of 110 sampling days (IQR: 60–155) at each location. Samples were primarily collected during the spring, summer, and autumn months with 1–7 integrated filter samples collected per site (the number of days varied across sites depending on how often TEOM filters were changed throughout the year, typically every 6-weeks). An in vitro assay based on a synthetic respiratory tract lining fluid was used to quantify regional OP^GSH^ and OP^AA^ (Additional file 2: Supplemental methods). In this study, the term *oxidative potential* is used to describe the ability of regional PM_2.5_ filter extracts to deplete glutathione (OP^GSH^) and ascorbate (OP^AA^) in the synthetic respiratory tract lining fluid whereas *oxidative burden* is used to describe the product of daily PM_2.5_ mass concentrations and regional estimates of oxidative potential.

### Statistical analysis

Conditional logistic regression models were used to estimate the impact of ambient PM_2.5_ on the risk of emergency room visits for myocardial infarction. All models were adjusted for 3-day mean ambient temperature and relative humidity using restricted cubic splines with 3 equally spaced knots. All analyses pooled cases across cities in order to evaluate potential effect modification by regional differences in oxidative potential; a cluster variance estimator was used to account for potential within-city correlations. Four different exposure lag periods were evaluated for PM_2.5_: lag-0 (the same day as the emergency room visit), lag-1 (the day prior to the visit), lag-2 (two days prior to the visit), and the mean of lags 0–2 (i.e. 3-day mean). Since the case-crossover design compares cases to themselves at different points in time it adjusts for factors that do not vary within individuals over short time-periods (e.g. age, smoking status, body mass index). In this study, matched sets consisted of the case period (the day of the emergency room visit) and control periods selected on the same day of the week in the same month and year as the case period (i.e. 3–4 referent periods per case). This time-stratified approach to referent selection has been shown to result in unbiased conditional logistic regression estimates in case-crossover studies [19]. Two different approaches were used to evaluate the impact of regional oxidative potential on the relationship between ambient PM_2.5_ and emergency room visits for myocardial infarction. First, *oxidative burden* metrics were generated by multiplying ambient PM_2.5_ mass concentrations by regional estimates of glutathione (OP^GSH^) and ascorbate-related (OP^AA^) oxidative potential. These parameters reflect a re-weighting of PM_2.5_ mass data according to regional oxidative potential and were treated as separate exposure variables in the analysis. Model AIC (Akaike Information Criterion) values were compared between PM_2.5_ mass and oxidative burden models to evaluate model fit. As a second approach, relationships between ambient PM_2.5_ mass concentrations and emergency room visits for myocardial infarction were examined across strata of regional OP^GSH^ and OP^AA^ (separately) and potential trends were examined across these strata. In particular, the statistical significance of interaction terms between PM_2.5_ and categorical variables for percentiles (<25^th^, 25-50^th^, >50-75^th^, >75^th^) of OP^GSH^ and OP^AA^ were examined. Potential effect modification by age and sex were evaluated through stratified analyses and by including the appropriate interaction terms in statistical models (age was treated as a continuous variable for interaction terms). As sensitivity analysis, we also examined the impact of adding ambient NO_2,_ O_3_, O_x_, or O_x_^wt^ to the PM_2.5_ models. The same lag-times as above were explored for these variables and the lag-time with the strongest association was used in sensitivity analyses. Potential three-way interactions between PM_2.5_, regional oxidative potential, and O_x_/O_x_^wt^ were also explored through stratified analyses and by including the appropriate interaction terms in statistical models (as continuous variables). Finally, since estimates of regional oxidative potential were not based on the same number of samples at each site, we repeated the main analysis for PM_2.5_ excluding sites with less than at least 1-month (30-days) of data (below the 10^th^ percentile of total days sampled across sites) to verify that these sites did not drive the overall results (Additional file 3: Figure S1). Risk estimates for PM_2.5_ are expressed per 5 μg/m^3^ change in ambient concentrations as this increment was approximately equal to the interquartile range of mean differences between case and control periods. All statistical analyses were conducted using Stata (version 13).

## Results

**Fig. 1:**
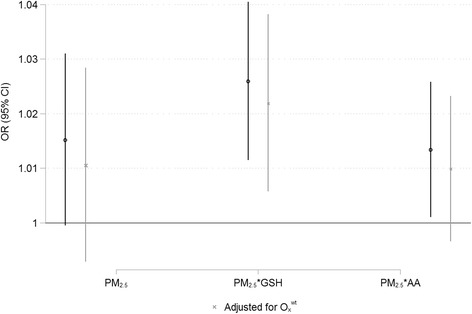
Lag-0 PM_2.5_, PM_2.5_ oxidative burden, and risk of MI Legend: Risk of emergency room visits for myocardial infarction associated with Lag-0 PM_2.5_ and PM_2.5_ oxidative burden in Ontario, Canada (2004–2011). Risk estimates reflect a 5 μg/m^3^ change in PM_2.5_ and a 1-unit change in PM_2.5_*GSH and PM_2.5_*AA. All models are adjusted for 3-day mean ambient temperature and relative humidity

**Fig. 2:**
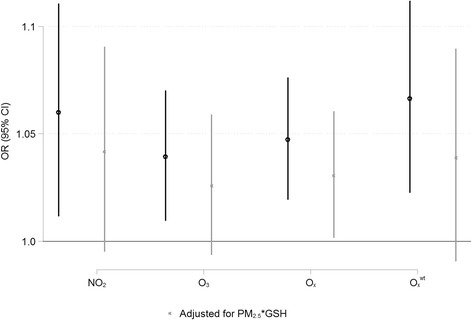
3-day mean NO_2_, O_3_, and combined oxidant capacity (O_x_ and O_x_^wt^) and risk of MI. Legend: Risk of emergency room visits for myocardial infarction associated with 3-day mean NO_2_, O_3_, and combined oxidant capacity (O_x_ and O_x_^wt^) in Ontario, Canada (2004–2011). Risk estimates reflect a 10 ppb change in ambient concentration and all models are adjusted for 3-day mean ambient temperature and relative humidity. Coefficients for NO_2_ and O_3_ are from two-pollutant models

**Fig. 3:**
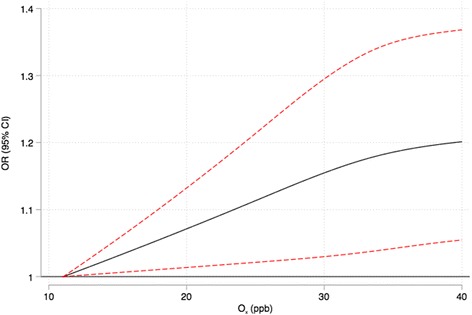
Concentration response plot for O_x_ and risk of MI. Legend: Concentration response plot for O_x_ (using cubic splines with 4 knots) and risk of emergency room visits for myocardial infarction adjusted for lag-0 PM_2.5_*GSH and 3-day mean ambient temperature and relative humidity

**Fig. 4:**
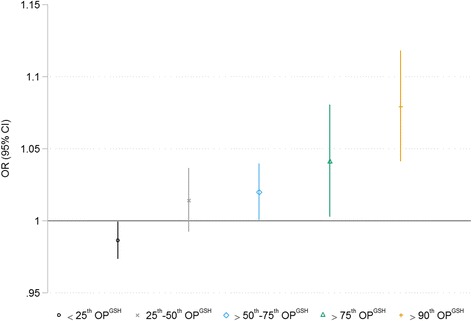
Lag-0 PM_2.5_ and risk of MI across strata of regional OP^GSH^ Legend: Ambient PM_2.5_ and risk of emergency room visits for myocardial infarction at different values of regional OP^GSH^. Risk estimates reflect a 5 μg/m^3^ change in Lag-0 PM_2.5_. All models are adjusted for 3-day mean ambient temperature and relative humidity

**Fig. 5:**
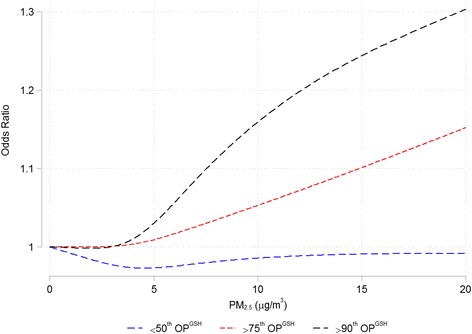
Concentration response plots for PM_2.5_ and risk of MI. Legend: Concentration response plots for PM_2.5_ and risk of emergency room visits for myocardial infarction below the 50^th^ percentile (blue) and above the 75^th^ (red line) and 90^th^ percentiles (black line) of OP^GSH^ using cubic splines with 4 knots. All models are adjusted for 3-day mean ambient temperature and relative humidity

In total, 30,101 cases of myocardial infarction were included in the analyses. Cases were predominately male (64 %) and had a mean age of 67.5 years. In general, daily PM_2.5_ mass concentrations were low (mean: 6.91 μg/m^3^) and oxidative potential varied substantially across sites with values ranging from 0.06–0.35 % depletion/μg PM_2.5_ for OP^GSH^ and 0.12–0.39 % depletion/μg PM_2.5_ for OP^AA^ (Additional file 1: Table S1; Additional file 3: Figure S2). Daily mean PM_2.5_ mass concentrations were similar across quartiles of oxidative potential (Additional file 3: Figure S3) and regional estimates of OP^GSH^ and OP^AA^ were weakly correlated with each other (*r* = −0.34) and were not correlated with the total number of sampling days at each site (*r* <0.20). Daily mean PM_2.5_ concentrations were weakly correlated with NO_2_ (r = 0.25), O_3_ (*r* = 0.15), O_x_ (*r* = 0.34), and O_x_^wt^ (*r* = 0.26); ambient NO_2_ and O_3_ were inversely correlated (*r* = −0.38). There was no correlation between the mean difference in PM_2.5_ concentrations between case and control days and regional estimates of OP^GSH^ (*r* = 0.02) or OP^AA^ (*r* = 0.007). In addition, the variance of this difference was similar across quartiles of oxidative potential suggesting that the power to detect an association did not vary across equally spaced quartiles of oxidative potential. The mean difference in PM_2.5_ concentrations between case and control days was 0.52 μg/m^3^ (95 % CI: −44.7, 51.7). Same day PM_2.5_ oxidative burden concentrations were associated with increased risks of hospitalization for myocardial infarction (Fig. 1). Risk estimates for exposures at lag-1 and lag-2 were lower than for lag-0 whereas risk estimates for 3-day mean exposure were similar to those for lag-0 (Additional file 1: Table S3). A comparison of AIC values across models indicated a better fit for glutathione-related oxidative burden (AIC = 87,710) compared to PM_2.5_ (AIC = 87,718) and ascorbate-related oxidative burden (AIC = 87,717). Significant effect modification by age or sex was not detected for any of the exposure variables (*p* > 0.05) although risks tended to be larger among men (Additional file 1: Tables S5-S6). The relationship between glutathione-related oxidative burden and myocardial infarction was robust to adjustment for 3-day mean ambient NO_2_, O_3_, O_x_, and O_x_^wt^ (Additional file 1: Table S7); however, the coefficient for ascorbate-related oxidative burden was not significantly increased when these parameters were included in the model. Excluding sites with less than 1-month of oxidative potential data did not change these results (Additional file 3: Figure S4). Coefficients for ambient NO_2_ and O_3_ were larger in two-pollutant models (i.e. containing both NO_2_ and O_3_) than in single-pollutant models (Additional file 1: Table S4; Additional file 3: Figure S5) but these pollutants were not associated with emergency room visits for myocardial infarction when models were adjusted for glutathione-related oxidative burden (Fig. 2). Conversely, the relationship between 3-day mean O_x_ and emergency room visits for myocardial infarction was robust to adjustment for glutathione-related oxidative burden with a linear increase in risk observed over a broad concentration range (Figure 3). In general, stronger associations were observed for O_x_^wt^ compare to O_x_ but these estimates tended to be less precise. Regional differences in glutathione-related oxidative potential modified the impact of ambient PM_2.5_ on the risk of myocardial infarction (*p* = 0.026) with stronger associations observed in regions with higher OP^GSH^ (Figs. 4–5) (Additional file 3: Figure S6). Regional OP^AA^ did not modify the impact of ambient PM_2.5_ on the risk of emergency room visits for myocardial infarction (*p* = 0.164) (Additional file 1: Table S8) but a significant three-way interaction was detected between PM_2.5_, OP^GSH^, and O_x_^wt^ (*p* < 0.001 for the continuous interaction term). Specifically, the strongest associations between ambient PM_2.5_ and emergency room visits for myocardial infarction occurred in areas with high glutathione-related oxidative potential and high redox-weighted oxidant capacity of NO_2_ and O_3_ (Table 1). A similar interaction was also observed for O_x_ (Additional file 1: Table S9).

**Table 1:** Percent change (95 % CI) in risk of emergency room visits for myocardial infarction associated with Lag-0 PM_2.5_ across strata of glutathione-related oxidative potential (OP^GSH^) and combined redox-weighted oxidant capacity of NO_2_ and O_3_ (O_x_^wt^)

Percentile of 3-day mean O_x_^wt^	Percentile of Regional OP^GSH^			
	≤50^th^	>50^th^	>75^th^	>90^th^
≤50^th^	−2.0 (−5.0, 1.0)	0.57 (−4.0, 5.0)	2.9 (−1.2, 7.2)	6.0 (0.0, 13)
>50^th^	1.5 (−0.6, 3.6)	5.5 (3.0, 8.0)	6.4 (2.0, 11)	10 (7.5, 13)
>75^th^	1.5 (−1.3, 4.4)	5.7 (2.5, 9.1)	6.7 (1.8, 12)	13 (6.8, 19)
>90^th^	1.4 (−4.6, 7.8)	9.0 (3.5, 15)	9.2 (0.46, 19)	29 (26, 33)

Risk estimates reflect a 5 μg/m^3^ change in PM_2.5_. All models are adjusted for 3-day mean ambient temperature and relative humidity (cubic splines)

## Discussion

In this study, we examined the impact of regional differences in the oxidative properties of PM_2.5_ on the relationship between PM_2.5_ and emergency room visits for myocardial infarction. In general, our findings suggest that ambient PM_2.5_ is associated with an increased risk of myocardial infarction but that regional differences in glutathione-related oxidative potential modify this risk. Moreover, our results suggest that the combined oxidant capacity of NO_2_ and O_3_ may be more strongly associated with myocardial infarction than either pollutant on its own and may further amplify the risk of myocardial infarction associated with PM_2.5_. Indeed, the combined oxidant capacity of NO_2_ and O_3_ was associated with myocardial infarction even after adjusting for PM_2.5_ oxidative burden whereas risk estimates for NO_2_ and O_3_ were not significantly increased after adjusting for this parameter. While other studies have reported increased risks of myocardial infarction with short-term changes in ambient PM_2.5_ [1–5], this is the first study to report significant effect modification by regional differences in PM_2.5_ oxidative potential. Indeed, this finding may have several important implications with respect to understanding and ultimately decreasing the public health impacts of PM_2.5_. In particular, our results suggest that the acute cardiovascular health risks of ambient PM_2.5_ may not be adequately characterized by mass concentrations alone. Moreover, regional estimates of glutathione-related oxidative potential may help to prioritize risk management activities and/or identify regions with sources most relevant to cardiovascular health. In doing so, expanded use of the combined oxidant capacity of NO_2_ and O_3_ may further facilitate this process as health risks may be greatest in areas with high oxidative potential and high O_x_/O_x_^wt^. This may be particularly helpful in countries, like Canada, where large geographic areas have similar PM_2.5_ mass concentrations but regional differences in sources may contribute to differences in particle toxicity.

Glutathione s-transferase (GST) enzymes are important for anti-oxidant defence as they act to neutralize oxidative stress by catalyzing the conjunction of reactive oxygen species with reduced glutathione [22]. Polymorphisms in these genes are common [23] and may modify the acute cardiovascular health effects of air pollution [6]. In particular, a recent study reported a stronger association between same-day PM_2.5_ and ventricular arrhythmias among subjects with GST gene polymorphisms that decrease anti-oxidant capacity [12]. Moreover, others have reported stronger decreases in heart rate variability (a measure of autonomic function) in response to second hand smoke [24] and ambient PM_2.5_ [10, 11] among subjects with reduced anti-oxidant capacity owing to polymorphisms in GST genes. These findings support the biological plausibility of effect modification by regional difference in glutathione-related oxidative potential as altered cardiac autonomic modulation is one pathway through which particulate air pollution is thought to contribute to acute cardiovascular morbidity [25]. Moreover, the fact that the combined oxidant capacity of NO_2_ and O_3_ magnified the impact of PM_2.5_ on the risk of myocardial infarction (particularly in areas with high oxidative potential) lends further support to the crucial role of oxidative stress in air pollution health effects. Ascorbate is also an important anti-oxidant in the body and some evidence suggests that plasma ascorbate concentrations are inversely correlated with cardiovascular mortality [26] and blood pressure [27]. Moreover, low plasma ascorbate concentrations have been associated with an increased risk of myocardial infarction [28]. In this study, ascorbate-related oxidative potential did not have a strong impact on the relationship between ambient PM_2.5_ and emergency room visits for myocardial infarction and the reasons for this are not entirely clear. One explanation may be that dietary intake of ascorbate offsets any depletion caused by inhaled PM_2.5_. Alternatively, ascorbate depletion in the lung lining fluid may not translate into lower plasma ascorbate concentrations which for ascorbate may be a more important marker of overall anti-oxidant capacity. Future studies should aim to clarify this point as our results suggest that specific components/sources of PM_2.5_ may differentially impact the depletion of ascorbate and glutathione which in turn may translate into differential toxicity with respect to cardiovascular health. To our knowledge only one other study has examined the acute health impacts of the combined oxidant capacity of NO_2_ and O_3_ [9]. Specifically, Williams et al. conducted a time-series study of daily morality in London, UK and observed a stronger association between 24-h O_x_ and daily mortality compared to single pollutant models for NO_2_ and O_3_ [9]. These authors also reported that stronger (but less precise) associations were observed for the redox-weighted measure of oxidant capacity compared to O_x_. Moreover, Williams et al. observed that risk estimates for NO_2_ and O_3_ increased when both pollutants were included in the same model [9] and Jerrett et al. [29] reported a similar effect for cardiovascular and ischemic heart disease mortality in a longitudinal a study in California, USA. Our findings are consistent with these results as stronger associations were observed for O_x_ in single pollutant models (compared to NO_2_ and O_3_) and coefficients for NO_2_ and O_3_ increased when both pollutants were included in the same model. However, only O_x_ remained associated with myocardial infarction after controlling for glutathione-related oxidative burden suggesting that this measure may provide a more accurate reflection of health risks posed by mixtures of NO_2_ and O_3_ than either pollutant on its own or together in the same model. While this study had a number of important advantages including quantitative estimates of regional oxidative potential and a large number of cases it is important to note several limitations. First, since the duration of monitoring used to estimate regional oxidative potential varied across sites (and was largely limited to the spring, summer, and autumn months), exposure measurement error likely impacted the characterization of regional oxidative potential. However, sensitivity analyses excluding sites with the least amount of data did not have an important impact on the results and clear seasonal trends in oxidative potential have not been reported [30]. Nevertheless, regional estimates of oxidative potential were also collected after the case ascertainment period and thus a given region may have been misclassified with respect to oxidative potential if values changed substantially over time. This likely biased risk estimates for oxidative burden measures toward the null; however, non-differential error could have biased stratified analyses (across quartiles of oxidative potential) in either direction depending on the pattern of misclassification across strata; however, this is not a likely explanation for the observed trend in myocardial infarction risk across strata of glutathione-related oxidative potential. A further limitation is related to the fact that we did not have information on spatial differences in oxidative potential within cities and the use of fixed-site measures likely contributed to exposure misclassification for oxidative potential as noted above. Similarly, we did not have information on day-to-day changes in oxidative potential *within* regions and thus we could not evaluate how temporal changes in oxidative potential within a given area impact the strength of the association between ambient PM_2.5_ acute cardiorespiratory morbidity. However, the type error imparted by using regional estimates of OP as opposed to daily values was likely Berkson type error (i.e. true daily values distributed around the regional mean estimates) and this type of error is not expected to bias associations toward the null. Nevertheless, future studies should evaluate this question as the acute health risks of short-term exposure to ambient PM_2.5_ may be modified by both within and between city differences in oxidative potential. Finally, as this is the first study to examine the impact of regional differences in oxidative potential on the relationship between PM_2.5_ and myocardial infarction, we cannot rule out other unmeasured factors or chance as an explanation of our findings. However, systematic differences in human behaviour between cities (i.e. more/less time spent outdoors) are not a likely explanation of our results as the cities included in our analyses share more or less the same climate and housing characteristics. A more important question relates to potential differences in local sources between regions and how these differences may translate into differences in PM_2.5_ oxidative potential and overall particle toxicity. This question needs to be addressed and may provide important information for future risk management activities aimed at reducing the public health impacts of particulate air pollution.

## Conclusions

Regional differences in glutathione-related oxidative potential may modify the impact of PM_2.5_ mass concentrations on the risk of myocardial infarction with the strongest associations observed in regions with the highest oxidative potential. Moreover, the combined oxidative capacity of NO_2_ and O_3_ may magnify this effect. These measures may provide an additional means of prioritizing risk management activities aimed at reducing the public health impacts of particulate air pollution.

## Additional files

Additional file 1: (**Table S1**: Descriptive statistics for myocardial infarction cases in Ontario, Canada; **Table S2**: Daily concentrations of ambient air pollutants in Ontario, Canada (2004–2011); **Table S3**: Percent change in risk of emergency room visits for myocardial infarction with PM_2.5_ and PM_2.5_ oxidative burden in Ontario, Canada (2004–2011); **Table S4**: Percent change in risk of emergency room visits for myocardial infarction associated with 3-day mean NO_2_, O_3_, and O_x_ Ontario, Canada (2004–2011); **Table S5**: Percent change in risk of emergency room visits for myocardial infarction with lag-0 PM_2.5_ and PM_2.5_ oxidative burden in Ontario, Canada (2004–2011): evaluation of effect modification by gender; **Table S6**: Percent change in risk of emergency room visits for myocardial infarction with PM_2.5_ and PM_2.5_ oxidative burden in Ontario, Canada (2004–2011): evaluation of effect modification by age; **Table S7**: Percent change in risk of emergency room visits for myocardial infarction associated with Lag-0 PM_2.5_ and PM_2.5_ oxidative burden in Ontario, Canada (2004–2011) with sensitivity analyses including additional adjustment for 3-day mean ambient NO_2_, O_3,_ O_x_, or O_x_^wt^; **Table S8**: Impact of regional PM_2.5_ oxidative potential on the relationship between ambient PM_2.5_ and emergency room visits for myocardial infarction; **Table S9**: Percent change (95 % CI) in risk of emergency room visits for myocardial infarction associated with PM_2.5_ across strata of regional OP^GSH^ and daily O_x_). (PDF 430 kb) Additional file 2: Supplemental methods. (PDF 308 kb) Additional file 3: (**Figure S1**: Total sampling days for regional oxidative potential (OP) at each site (2012–2013). Values below the 10^th^ percentile (30 days, indicated by the red vertical line) were excluded for sensitivity analyses; **Figure S2**: Regional estimates of glutathione (OP^GSH^) and ascorbate (OP^AA^)-related oxidative potential in Ontario, Canada (2012–2013); **Figure S3**: Distribution of daily PM_2.5_ mass concentrations across quartiles of glutathione-related oxidative potential (OP^GSH^); **Figure S4**: Ambient PM_2.5,_ PM_2.5_ oxidative burden and emergency room visits for myocardial infarction: sensitivity analyses excluding sites with less than 1-month of oxidative potential data; **Figure S5**: Concentration response plots (using cubic splines with 4 knots) for NO_2_, O_3_, and O_x_ and risk of emergency room visits for myocardial infarction; **Figure S6**: Concentration response plots for PM_2.5_ and risk of emergency room visits for myocardial infarction above the 90^th^ percentile of OP^GSH^ and below the 50^th^ percentile using cubic splines with 4 knots). (PDF 382 kb)

## Abbreviations

AIC: Akaike Information Criterion
CIHI: Canadian Institute for Health Information
GST: glutathione-s-transferase
NACRS: National Ambulatory Care Reporting System.
NAPS: National Air Pollution Surveillance
OP^AA^: ascorbate-related oxidative potential
OP^GSH^: glutathione-related oxidative potential
O_x_: oxidant capacity of NO_2_ and O_3_
O_x_^wt^: redox-weighted oxidant capacity of NO_2_ and O_3_

## References

[1] Rosenthal FS, Kuisma M, Lanki T, Hussein T, Boyd J, Halonen JI, Pekkanen J. Association of ozone and particulate air pollution with out-of-hospital cardiac arrest in Helsinki, Finland: evidence of two different etiologies. J Expo Sci Environ Epidemiol. 2013;23(3):281–8. 10.1038/jes.2012.121 23361443

[2] Gardner B, Ling F, Hopke PK, Frampton WM, Utell MJ, Zareba W, Cameron SJ, Chalupa D, Kane C, Kulandaisamy S, Topf MC, Rich DQ. Ambient fine particulate air pollution triggers ST-elevation myocardial infarction, but not non-ST elevation myocardial infarction : a case-crossover study. Part Fibre Toxicol. 2014;11:1. 10.1186/1743-8977-11-1 PMC3891992 24382024

[3] Rich DQ, Kipen HM, Zhang J, Kamat L, Wilson AC, Kostis JB (2010). Triggering of transmural infarctions, but not nontransmural infarctions, by ambient fine particles. Environ Health Perspect.

[4] Dominici F, Peng RD, Bell ML, Pham L, McDermott A, Zeger SL, Samet JM. Fine particulate air pollution and hospital admission for cardiovascular and respiratory diseases. JAMA. 2006;295(10):1127–34. 10.1001/jama.295.10.1127 PMC3543154 16522832

[5] Belleudi V, Faustini A, Stafoggia M, Cattani G, Marconi A, Perucci CA, Forastiere F. Impact of fine and ultrafine particles on emergency hospital admissions for cardiac and respiratory diseases. Epidemiology. 2012;21(3):414–23. 10.1097/EDE.0b013e3181d5c021 20386174

[6] Weichenthal SA, Godri-Pollitt K, Villeneuve PJ (2013). PM2.5, oxidant defence and cardiorespiratory health: a review. Environ Health.

[7] Li N, Hao M, Phalen RF, Hinds WC, Nel AE (2003). Particulate air pollutants and asthma: a paradigm for the role of oxidative stress in PM-induced adverse health effects. Clin Immunol.

[8] Kelly FJ (2003). Oxidative stress: its role in air pollution and adverse health effects. Occup Environ Med.

[9] Williams ML, Atkinson RW, Anderson R, Kelly FJ (2014). Associations between daily mortality in London and combined oxidant capacity, ozone and nitrogen dioxide. Air Qual Atmos Health.

[10] Schwartz J, Park SK, O’Neil MS, Vokonas PS, Sparrow D, Weiss S, Kelsey K. Glutathione-S-transferase M1, obesity, statins, and autonomic effects of particles. Am J Respir Crit Care Med. 2005;172(12):1529–33. 10.1164/rccm.200412-1698OC PMC2718454 16020798

[11] Chahine T, Baccarelli A, Litonjua A, Wright RO, Suh H, Gold DR, Sparow D, Vokonas P, Schwartz J. Particulate air pollution, oxidative stress genes, and heart rate variability in an elderly cohort. Environ Health Perspect. 2007;115(11):1617–22. 10.1289/ehp.10318 PMC2072834 18007994

[12] Zanobetti A, Coull BA, Gryparis A, Kloog I, Sparrow D, Vokonas PS, Wright RO, Gold DR, Schwartz J. Associations between arrhythmia episodes and temporally and spatially resolved black carbon and particulate matter in elderly patients. Occup Environ Health. 2014;71(3):201–7. 10.1136/oemed-2013-101526 PMC4371778 24142987

[13] Künzli N, Mudway IS, Gotschi T, Shi T, Kelly FJ, Cook S, Burney P, Forsberg B, Gauderman JW, Hazenkamp ME, Heinrich J, Jarvis D, Norback D, Payo-Losa F, Poli A, Sunyer J, Borm PJ. Comparison of oxidative properties, light absorbance, and total and elemental mass concentration of ambient PM2.5 collected at 20 European sites. Environ Health Perspect. 2006;114(5):684–90. 10.1289/ehp.8584 PMC1459920 16675421

[14] Janssen NAH, Yang A, Strak M, Steenhof M, Hellack B, Gerlofs-Nijland ME, Kuhlbusch T, Kelly F, Harrison R, Brunekreef B, Hoek G, Cassee F. Oxidative potential of particulate matter collected at sites with different source characteristics. Sci Total Environ. 2014;472:572–81. 10.1016/j.scitotenv.2013.11.099 24317165

[15] Yang A, Wang M, Eeftens M, Beelen R, Dons E, Leseman DL, Brunekreef B, Cassee FR, Janssen NA, Hoek G. Spatial variations and land use regression modeling of the oxidative potential of fine particles. Environ Heath Perspect. 2015. April 3. [Epub ahead of print]. 10.1289/ehp.1408916 PMC4629740 25840153

[16] Yanosky JD, Tonne CC, Beevers SD, Wilkinson P, Kelly FJ (2012). Modeling exposures to the oxidative potential of PM10. Environ Sci Technol.

[17] Godri KJ, Duggan ST, Fuller GW, Baker T, Green D, Kelly FJ, Mudway IS. Particulate matter oxidative potential from waste transfer station activity. Environ Health Perspect. 2010;118(4):493–8. 10.1289/ehp.0901303 PMC2854725 20368130

[18] Ayres JG, Borm P, Cassee FR, Castranova V, Donaldson K, Ghio A, Harrison RM, Hider R, Kelly F, Kooter IM, Marano RL, Mudway I, Nel A, Sioutas C, Smith S, Baeza-Squiban A, Cho A, Duggan S, Froines J. Evaluating the toxicity of airborne particulate matter and nanoparticles by measuring oxidative stress potential-a workshop report and concensus statement. Inhal Toxicol. 2008;20(1):75–99. 10.1080/08958370701665517 18236225

[19] Janes H, Sheppard L, Lumley T (2005). Case-crossover analyses of air pollution exposure data: referent selection strategies and their implications for bias. Epidemiology.

[20] Gibson D, Richards H, Chapman A (2008). The national ambulatory care reporting system: factors that affect the quality of its emergency data. Int J Information Quality.

[21] Bratsch SG. Standard electrode potentials and temperature coefficients in water at 298.15 K. Vol. 18, No.1, 1-21. Available at: http://www.nist.gov/data/PDFfiles/jpcrd355.pdf.

[22] Hayes JD, McLellan LI (1999). Glutathione and glutathione-dependent enzymes represent a co-ordinately regulated defense against oxidative stress. Free Rad Biol Med.

[23] Minelli C, Wei I, Sagoo G, Jarvis D, Shaheen S, Burney P (2011). Interactive effects of antioxidant genes and air pollution on respiratory function and airway disease: a HuGE review. Am J Epidemiol.

[24] Probst-Hensch NM, Imboden M, Felber Dietrich D, Barthelemy JC, Ackermann-Liebrich U, Berger W, Gaspoz JM, Schwartz J. Glutathione s-transferase polymorphisms, passive smoking, obesity, and heart rate variability in non-smokers. Environ Health Perspect. 2008;116(11):1494–9. 10.1289/ehp.11402 PMC2592269 19057702

[25] Brook RD, Rajagopalan S, Pope 3rd CA, Brook JR, Bhatnager A, Diez-Roux AV, Holguin F, Hong Y, Luepker RV, Mittleman MA, Peters A, Siscovick D, Smith SC Jr, Whitsel L, Kaufman JD. AHA scientific statement: particulate matter air pollution and cardiovascular disease. Circulation. 2010;121(21):2331–78. 10.1161/CIR.0b013e3181dbece1 20458016

[26] Khaw KT, Bingham S, Welch A, Luben R, Wareham N, Oakes S, Day N. Relation between plasma ascorbic acid and mortality in men and women in EPIC-Norfolk prospective study: a prospective population study. European Prospective Investigation into Cancer and Nutrition. Lancet. 2001;357:657–63. 10.1016/s0140-6736(00)04128-3 11247548

[27] Myint PK, Luben RN, Wareham NJ, Khaw KT (2011). Association between plasma vitamin C concentrations and blood pressure in the European prospective investigation into cancer-Norfolk population-based study. Hypertension.

[28] Nyyssonen K, Parviainen MT, Salonen R, Tuomilehto J, Salonen JT (1997). Vitamin C deficiency and risk of myocardial infarction: prospective population study of men from eastern Finland. BMJ.

[29] Jerrett M, Burnett RT, Beckerman BS, Turner MC, Krewski D, Thurston G, Martin RV, van Donkelaar A, Hughes E, Shi Y, Gapstur SM, Thun MJ, Pope CA 3rd. Spatial analysis of air pollution and mortality in California. Am J Resp Crit Care Med. 2013;188(5):593–9. 10.1164/rccm.201303-0609OC PMC5447295 23805824

[30] Szigeti T, Ovari M, Dunster C, Kelly FJ, Lucarelli F, Zaray G (2015). Changes in chemical composition and oxidative potential of urban PM2.5 between 2010 and 2013 in Hungary. Sci Total Environ.

