# Supplementary material for: Ambient PM2.5 and risk of emergency room visits for myocardial infarction: impact of regional PM2.5 oxidative potential: a case-crossover study
Source: Environ Health. 2016 Mar 24;15:46. doi: 10.1186/s12940-016-0129-9 (PMC4806515; doi:10.1186/s12940-016-0129-9)
Supplement: Additional file 1: — (Table S1: Descriptive statistics for myocardial infarction cases in Ontario, Canada; Table S2: Daily concentrations of ambient air pollutants in Ontario, Canada (2004–2011); Table S3: Percent change in risk of emergency room visits for myocardial infarction with PM2.5 and PM2.5 oxidative burden in Ontario, Canada (2004–2011); Table S4: Percent change in risk of emergency room visits for myocardial infarction associated with 3-day mean NO2, O3, and Ox Ontario, Canada (2004–2011); Table S5: Percent change in risk of emergency room visits for myocardial infarction with lag-0 PM2.5 and PM2.5 oxidative burden in Ontario, Canada (2004–2011): evaluation of effect modification by gender; Table S6: Percent change in risk of emergency room visits for myocardial infarction with PM2.5 and PM2.5 oxidative burden in Ontario, Canada (2004–2011): evaluation of effect modification by age; Table S7: Percent change in risk of emergency room visits for myocardial infarction associated with Lag-0 PM2.5 and PM2.5 oxidative burden in Ontario, Canada (2004–2011) with sensitivity analyses including additional adjustment for 3-day mean ambient NO2, O3, Ox, or Ox wt; Table S8: Impact of regional PM2.5 oxidative potential on the relationship between ambient PM2.5 and emergency room visits for myocardial infarction; Table S9: Percent change (95 % CI) in risk of emergency room visits for myocardial infarction associated with PM2.5 across strata of regional OPGSH and daily Ox). (PDF 430 kb) [file 12940_2016_129_MOESM1_ESM.pdf]

ADDITIONAL FILE 1

Ambient $PM_{2.5}$ and Risk of Emergency Room Visits for Myocardial Infarction: Impact of Regional $PM_{2.5}$ Oxidative Potential: A Case-Crossover Study

Scott Weichenthal
Health Canada
269 Laurier Ave West, Ottawa, Ontario, Canada, K1A 0K9
Email: scott.weichenthal@hc-sc.gc.ca

Eric Lavigne
Health Canada
269 Laurier Ave West, Ottawa, Ontario, Canada, K1A 0K9
Email: eric.lavigne@hc-sc.gc.ca

Greg Evans
University of Toronto
200 College St, Toronto, Ontario, Canada, M5S 3E5
greg.evans@utoronto.ca

Krystal Pollitt
University of Massachusetts
686 North Pleasant Street, Amherst, Massachusetts, 01003, USA
kpollitt@umass.edu

Rick T Burnett
Health Canada
50 Colombine Driveway, Ottawa, Ontario, Canada, K1A 0K9
Email: rick.burnett@hc-sc.gc.ca

Correspondence*
Scott Weichenthal
269 Laurier Ave West, Ottawa, Ontario, Canada, K1A 0K9
Telephone: 613-948-7765; Fax: 613-954-7612; Email: scott.weichenthal@hc-sc.gc.ca

Supplemental Tables

Table S1. Descriptive statistics for myocardial infarction cases in Ontario, Canada (2004-2011)

Location	No. Cases	% Male	Mean Age (SD) (years)
All Sites Combined	30,101	64	67.5 (15)
Barrie	80		
Burlington	1593		
Chatham	736		
Cornwall	1006		
Guelph	394		
Hamilton-Downtown	1065		
Hamilton-Mountain	1117		
Hamilton-West	168		
Kingston	1109		
Kitchener	4803		
Mississauga	1578		
Oshawa	347		
Peterborough	2081		
Sarnia	494		
St Catherines	1393		
Sudbury	1566		
Thunder Bay	662		
Toronto-Downtown	3029		
Toronto-East	5027		
Toronto-North	1853		

Table S2. Daily concentrations of ambient air pollutants in Ontario (2004-2011)

Air Pollutant	Mean (SD)	Median (IQR)	Range
$PM_{2.5}$ (µg/m^3)	6.91 (5.97)	5.12 (5.75)	<1- 56.8
$PM_{2.5}$*GSH (% Depletion/m^3)	1.10 (1.19)	0.71 (1.01)	<1-15.7
$PM_{2.5}$*AA (% Depletion/m^3)	1.63 (1.51)	1.17 (1.38)	<1-18.0
NO_2 (ppb)	12.3 (7.63)	10.8 (10.3)	<1- 65
O_3 (ppb)	25.7 (10.3)	25.1 (14.2)	<1 – 79.5
O_x (ppb)	38.0 (10)	37.2 (13)	9.6-88
O_x^{wt} (ppb)	21.2 (6.3)	20.7 (8.6)	4.5-54
Temperature (^{o}C)	8.05 (10.4)	8.60 (16.8)	-29 – 31.5
Relative Humidity (%)	74.3 (11.9)	75.0 (16.4)	27-100

$PM_{2.5}$*GSH, glutathione-related oxidative burden; $PM_{2.5}$ *AA, ascorbate-related oxidative burden; O_x, sum of NO_2 and O_3); O_x^{wt}, redox-weighed average of NO_2 and O_3; IQR, inter-quartile range.

Table S3. Percent change in risk of emergency room visits for myocardial infarction with $PM_{2.5}$ and $PM_{2.5}$ oxidative burden in Ontario, Canada (2004-2011)

Outcome	Lag	Exposure		
		$PM_{2.5}$	$PM_{2.5}$*GSH	$PM_{2.5}$*AA
		% Change (95% CI)	% Change (95% CI)	% Change (95% CI)
Myocardial Infarction	0	1.5 (-0.10, 3.1)	2.6 (1.1, 4.1)	1.3 (0.1, 2.6)
	1	0.90 (-0.005, 2.3)	1.5 (0.0, 3.2)	0.70 (-0.005, 1.9)
	2	0.30 (-0.01, 1.6)	0.30 (-0.012, 1.8)	0.30 (-0.010, 1.6)
	3-day mean	1.7 (-0.006, 4.1)	2.6 (0.2, 5.0)	1.5 (-0.006, 3.6)

Risk estimates reflect a 5 $\mu g/m^3$ change in $PM_{2.5}$ and a 1-unit change in $PM_{2.5}$*GSH and $PM_{2.5}$*AA. All models are adjusted for 3-day mean ambient temperature and relative humidity (cubic splines).

Table S4. Percent change in risk of emergency room visits for myocardial infarction associated with 3-day mean NO_2, O_3, and O_x Ontario, Canada (2004-2011).

Models	Percent Change (95% CI)	Adjusted for Lag-0 $PM_{2.5}$	Adjusted for Lag-0 $PM_{2.5}$*GSH
		Percent Change (95% CI)	Percent Change (95% CI)
Single Pollutant Models			
NO_2	4.4 (-0.30, 9.2)	3.7 (-0.70,8.4)	2.8 (-1.6, 7.3)
O_3	2.2 (-0.80, 5.2)	1.3 (-1.8, 4.6)	1.2 (-1.9, 4.4)
Two Pollutant Models			
NO_2	6.0 (1.1, 11)	5.7 (0.64, 11)	4.2 (-0.50, 9.1)
O_3	3.9 (0.94, 7.0)	3.2 (-0.020, 6.9)	2.6 (-0.70, 5.9)
Combined Oxidant Model			
O_x	4.7 (1.9, 7.6)	3.9 (0.65, 7.3)	3.1 (0.15, 6.1)
O_x-weighted	6.6 (2.2, 11)	4.7 (-0.60, 10)	3.9 (-0.01, 9.0)

AIC, Akaike Information Criterion; O_x, sum of NO_2 and O_3; O_x^{wt}, redox-weighed average of NO_2 and O_3. All risk estimates reflect a 10 ppb change in exposure. All models are adjusted for 3-day mean ambient temperature and relative humidity (cubic splines).

Table S5. Percent change in risk of emergency room visits for myocardial infarction with lag-0 $PM_{2.5}$ and $PM_{2.5}$ oxidative burden in Ontario, Canada (2004-2011): evaluation of effect modification by gender

Exposure	Gender		Percent Change (95% CI)
$PM_{2.5}$	Overall		1.5 (-0.10, 3.1)
		Men	2.1 (-0.10, 4.4)
		Women	0.41 (-2.0, 3.2)
			p=0.350
$PM_{2.5}$*GSH	Overall		2.6 (1.1, 4.1)
		Men	3.6 (1.2, 5.9)
		Women	0.93 (-2.0, 4.3)
			p=0.385
$PM_{2.5}$*AA	Overall		1.3 (0.10, 2.6)
		Men	1.9 (0.12, 3.7)
		Women	0.41 (-2.0, 2.5)
			p=0.455

Risk estimates reflect a 5 $\mu g/m^3$ change in $PM_{2.5}$ and a 1-unit change in $PM_{2.5}$*GSH and $PM_{2.5}$*AA. All models are adjusted for 3-day mean ambient temperature and relative humidity (cubic splines).

Table S6. Percent change in risk of emergency room visits for myocardial infarction with $PM_{2.5}$ and $PM_{2.5}$ oxidative burden in Ontario, Canada (2004-2011): evaluation of effect modification by age

Outcome	Exposure Lag	Age (years)	Exposure<br>$PM_{2.5}$<br>% Change (95% CI)	 $PM_{2.5}$*GSH<br>% Change (95% CI)	 $PM_{2.5}$*AA<br>% Change (95% CI)
Myocardial Infarction	Lag-0	All	1.5 (-0.10, 3.1)	2.6 (1.1, 4.1)	1.3 (0.1, 2.6)
		<56	3.3 (0.61, 6.1)	4.5 (1.3, 7.9)	3.3 (0.81, 5.8)
		57-68	0.28 (-3.1, 3.7)	1.8 (-1.1, 4.8)	0.31 (-2.6, 3.3)
		69-79	1.9 (-1.2, 5.1)	3.4 (-0.4, 7.4)	1.7 (-0.70, 4.0)
		>79	0.79 (-1.6, 3.2)	1.1 (-1.9, 4.1)	0.51 (-1.3, 2.3)
			p=0.664	p=0.448	p=0.404

Risk estimates reflect a 5 µg/m^3 change in $PM_{2.5}$ and a 1-unit change in $PM_{2.5}$*GSH and $PM_{2.5}$*AA. All models are adjusted for 3-day mean ambient temperature and relative humidity (cubic splines).

Table S7. Percent change in risk of emergency room visits for myocardial infarction associated with Lag-0 $PM_{2.5}$ and $PM_{2.5}$ oxidative burden in Ontario, Canada (2004-2011) with sensitivity analyses including additional adjustment for 3-day mean ambient NO_2, O_3, O_x, or O_x^{wt}

Exposure	Covariate added	Percent Change (95% CI)
$PM_{2.5}$	Base model	1.5 (-0.10, 3.1)
	NO_2	0.8 (-0.60, 2.1)
	O_3	1.4 (-0.20 3.0)
	NO_2 and O_3	0.29 (-1.3, 1.9)
	O_x	0.59 (-1.2, 2.4)
	O_x^{wt}	1.1 (-0.7, 2.8)
$PM_{2.5}$*GSH	Base model	2.6 (1.1, 4.1)
	NO_2	2.0 (0.5, 3.6)
	O_3	2.4 (0.8, 4.1)
	NO_2 and O_3	1.7 (0.010, 3.4)
	O_x	1.8 (0.16, 3.6)
	O_x^{wt}	2.2 (0.57, 3.8)
$PM_{2.5}$*AA	Base model	1.3 (0.1, 2.6)
	NO_2	0.8 (-0.30, 1.8)
	O_3	1.2 (-0.20, 2.6)
	NO_2 and O_3	0.43 (-0.70, 1.6)
	O_x	0.64 (-0.70, 2.0)
	O_x^{wt}	1.0 (-0.30, 2.3)

O_x, sum of NO_2 and O_3; O_x^{wt}, redox-weighed average of NO_2 and O_3. Risk estimates reflect a 5 $\mu g/m^3$ change in $PM_{2.5}$ and a 1-unit change in $PM_{2.5}$*GSH and $PM_{2.5}$*AA. All models are adjusted for 3-day mean ambient temperature and relative humidity (cubic splines).

Table S8. Impact of regional $PM_{2.5}$ oxidative potential on the relationship between ambient $PM_{2.5}$ and hospitalization for myocardial infarction and asthma

Exposure	Percentile of Oxidative Potential				
	% Change (95% CI)				
	< 25th	25-50th	>50-75th	>75th	> 90th
OP^{GSH}					
Lag0 $PM_{2.5}$	-1.4 (-2.7, 0.0)	1.4 (-0.08, 3.6)	2.0 (0.0060, 3.9)	4.1 (0.26, 8.0)	7.9 (4.1, 12)
OP^{AA}					
Lag0 $PM_{2.5}$	2.5 (-0.50, 5.6)	-1.0 (-2.7, 0.76)	1.5 (-0.90, 4.0)	2.8 (-0.80, 6.6)	0.87 (-1.6, 3.3)

Risk estimates reflect a 5 μg/m^3 change in $PM_{2.5}$. All models are adjusted for 3-day mean ambient temperature and relative humidity (cubic splines).

Table S9. Percent change (95% CI) in risk of emergency room visits for myocardial infarction associated with $PM_{2.5}$ across strata of regional OP^{GSH} and daily O_x

Percentile of 3-day mean O_x	Percentile of Regional OP^{GSH}			
	≤50th	> 50th	> 75th	> 90th
≤50th	-1.1 (-4.3, 2.2)	0.95 (-3.9, 6.0)	3.8 (-2.4, 11)	8.8 (-1.3, 20)
> 50th	1.7 (-0.10, 3.5)	4.8 (1.9, 7.7)	5.6 (0.49, 11)	11 (8.0, 13)
> 75th	3.2 (-0.80, 7.3)	6.7 (2.7, 11)	8.2 (1.1, 16)	17 (10, 24)
> 90th	0.90 (-4.6, 6.8)	6.3 (-1.2, 14)	3.8 (-1.6, 9.4)	16 (6.0, 26)

Risk estimates reflect a 5 $\mu g/m^3$ change in $PM_{2.5}$. All models are adjusted for 3-day mean ambient temperature and relative humidity (cubic splines).
