# Supplementary material for: Ambient PM2.5 and risk of emergency room visits for myocardial infarction: impact of regional PM2.5 oxidative potential: a case-crossover study
Source: Environ Health. 2016 Mar 24;15:46. doi: 10.1186/s12940-016-0129-9 (PMC4806515; doi:10.1186/s12940-016-0129-9)
Supplement: Additional file 2: — Supplemental methods. (PDF 308 kb) [file 12940_2016_129_MOESM2_ESM.pdf]

ADDITIONAL FILE 2

# Ambient $PM_{2.5}$ and Risk of Emergency Room Visits for Myocardial Infarction: Impact of Regional $PM_{2.5}$ Oxidative Potential: A Case-Crossover Study

Scott Weichenthal
Health Canada
269 Laurier Ave West, Ottawa, Ontario, Canada, K1A 0K9
Email: scott.weichenthal@hc-sc.gc.ca

Eric Lavigne
Health Canada
269 Laurier Ave West, Ottawa, Ontario, Canada, K1A 0K9
Email: eric.lavigne@hc-sc.gc.ca

Greg Evans
University of Toronto
200 College St, Toronto, Ontario, Canada, M5S 3E5
greg.evans@utoronto.ca

Krystal Pollitt
University of Massachusetts
686 North Pleasant Street, Amherst, Massachusetts, 01003, USA
kpollitt@umass.edu

Rick T Burnett
Health Canada
50 Colombine Driveway, Ottawa, Ontario, Canada, K1A 0K9
Email: rick.burnett@hc-sc.gc.ca

**Correspondence***
Scott Weichenthal
269 Laurier Ave West, Ottawa, Ontario, Canada, K1A 0K9
Telephone: 613-948-7765; Fax: 613-954-7612; Email: scott.weichenthal@hc-sc.gc.ca

## Supplemental Methods

### Laboratory Analysis of Regional Oxidative Potential

Integrated $PM_{2.5}$ samples were equilibrated for 24 hours in a weighing room with temperature (18-22 °C) and humidity (45-50%) controls. Filters were weighed pre- and post-extraction using a Sartorius model MC5 microbalance and PM was extracted from filter samples into HPLC grade methanol through sonication with a titanium probe sonicator operated at an amplitude of 5 microns for 30 seconds. Filters were then rinsed with additional methanol and sample extracts were dried with nitrogen gas in a water bath heated to 37 °C. Samples were re-suspended in a 5% methanol/ultrapure water solution to 75 μg/mL. The extraction efficiency for each filter sample was calculated by comparing station- reported filter masses with the net loss of filter mass from the filters, pre- to post-extraction. The median extraction efficiency was 97%.

Re-suspended $PM_{2.5}$ samples were incubated with a synthetic human respiratory tract lining fluid for 4 hours at 37 °C (1-2). This fluid was a 200 μM composite solution of physiologically-relevant antioxidants including ascorbate (AA), urate, and glutathione (GSH). Glutathione and ascorbate depletion were used to measure regional $PM_{2.5}$ oxidative potential expressed on a per mass basis (% depletion/μg). This operationally defined metric described the portion of the initial 200 μM reacted over four hours normalized by the 75 μg/mL $PM_{2.5}$ used in the assay. Urate was not included as PM does not have an important impact on urate depletion (2-3). The plate was incubated at 37 °C in a UV-vis plate reader (Molecular Devices, SpectraMax 190). Ascorbate-related oxidative potential was measured using absorbance spectra and percent ascorbate depletion was measured as the percent change in absorbance at a wavelength of 260 nm over the 4-hour incubation period.

After the 4-hour incubation period, the remaining reduced glutathione concentration was determined using the oxidized glutathione -reductase-5,5′-dithio-bis(2-nitrobenzoic acid)

recycling assay (4). Glutathione-related oxidative potential was expressed as the percent change between each sample and a 4-hour incubated water blank. $PM_{2.5}$ samples and controls were analyzed in triplicate on a 96-well plate at a final concentration of 75 μg PM/mL. Known positive (non-ferrous dust, NRC PD-1) and negative (carbon black, Arosperse 15B) controls were run in parallel with $PM_{2.5}$ samples to evaluate inter-experimental standardization .

**Supplemental References**


1. Godri KJ, Harrison RM, Evans T, Baker T, Dunster C, Mudway IS, et al. Increased oxidative burden associated with traffic component of ambient particulate matter at roadside and urban background schools sites in London. PloS ONE 2011; 6:e21961.
2. Mudway IS, Stenfors N, Duggan ST, Roxborough H, Zielinski H, Marklund SL, et al. An in vitro and in vivo investigation of the effects of diesel exhaust on human airway lining fluid antioxidants. Arch Biochem Biophys 2004; 423:200–212.
3. Künzli, N, Mudway IS, Botschi T, Shi T, Kelly FJ, Cook S, et al. Comparison of oxidative properties, light absorbance, and total and elemental mass concentration of ambient PM2.5 collected at 20 European sites. Environ Health Perspect 2006; 114: 684-690.
4. Baker MA, Cerniglia GJ, Zaman A. Microtiter plate assay for the measurement of glutathione and glutathione disulfide in large numbers of biological samples. Anal Biochem 1990; 190: 360-365.
