# Supplementary material for: Ambient PM2.5 and risk of emergency room visits for myocardial infarction: impact of regional PM2.5 oxidative potential: a case-crossover study
Source: Environ Health. 2016 Mar 24;15:46. doi: 10.1186/s12940-016-0129-9 (PMC4806515; doi:10.1186/s12940-016-0129-9)

ADDITIONAL FILE 3

Ambient $PM_{2.5}$ and Risk of Emergency Room Visits for Myocardial Infarction: Impact of Regional $PM_{2.5}$ Oxidative Potential: A Case-Crossover Study

Scott Weichenthal
Health Canada
269 Laurier Ave West, Ottawa, Ontario, Canada, K1A 0K9
Email: scott.weichenthal@hc-sc.gc.ca

Eric Lavigne
Health Canada
269 Laurier Ave West, Ottawa, Ontario, Canada, K1A 0K9
Email: eric.lavigne@hc-sc.gc.ca

Greg Evans
University of Toronto
200 College St, Toronto, Ontario, Canada, M5S 3E5
greg.evans@utoronto.ca

Krystal Pollitt
University of Massachusetts
686 North Pleasant Street, Amherst, Massachusetts, 01003, USA
kpollitt@umass.edu

Rick T Burnett
Health Canada
50 Colombine Driveway, Ottawa, Ontario, Canada, K1A 0K9
Email: rick.burnett@hc-sc.gc.ca

Correspondence*
Scott Weichenthal
269 Laurier Ave West, Ottawa, Ontario, Canada, K1A 0K9
Telephone: 613-948-7765; Fax: 613-954-7612; Email: scott.weichenthal@hc-sc.gc.ca

Supplemental Figures

Figure S1. Total sampling days for regional oxidative potential (OP) at each site (2012-2013). Values below the 10th percentile (30 days, indicated by the red vertical line) were excluded for sensitivity analyses

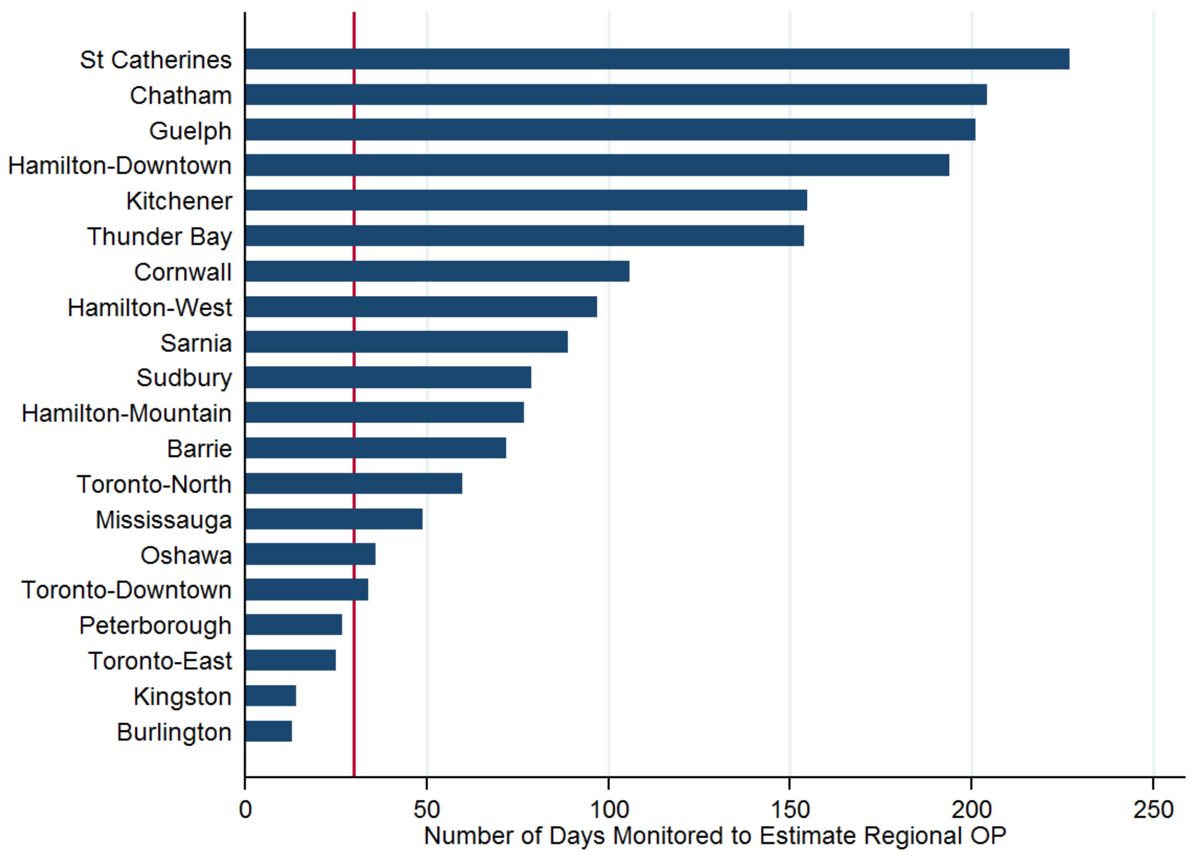

Figure S2. Regional estimates of glutathione (OP^{GSH}) and ascorbate (OP^{AA})-related oxidative potential in Ontario, Canada (2012-2013)

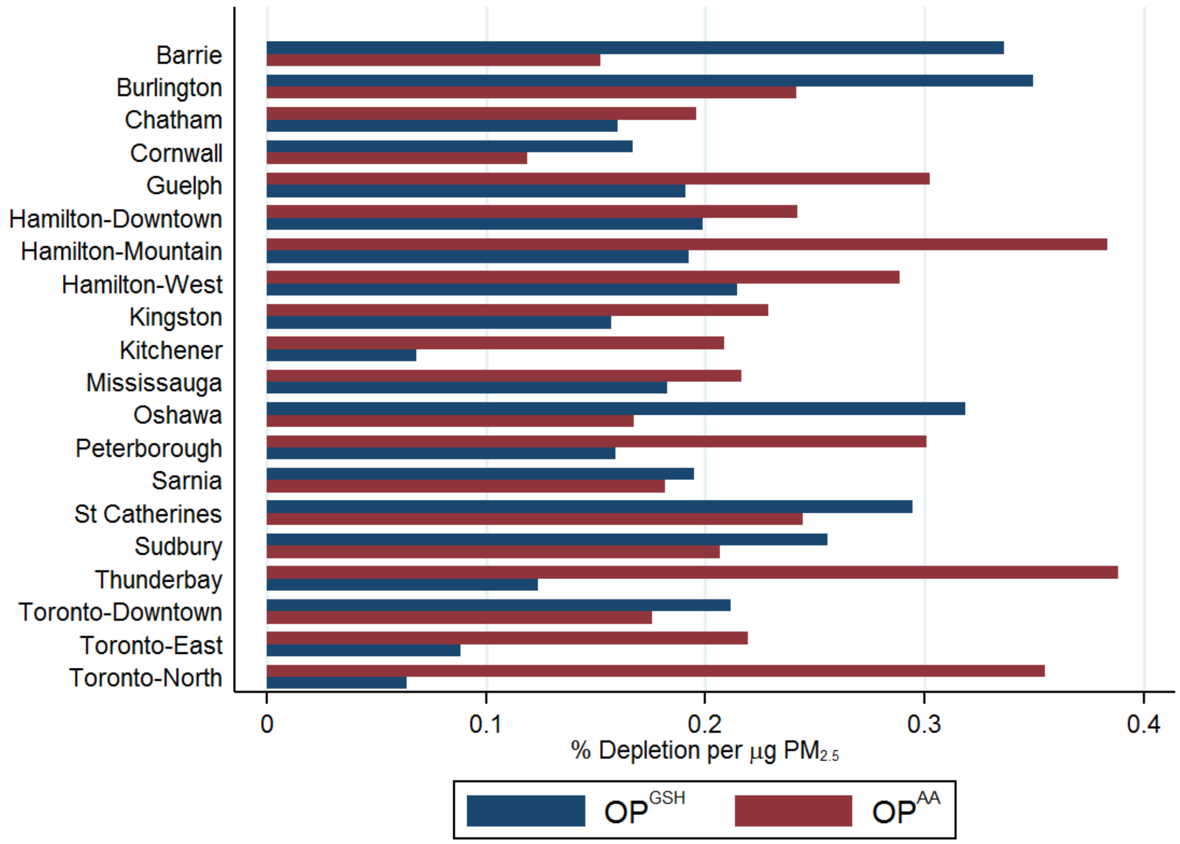

Figure S3. Distribution of daily $PM_{2.5}$ mass concentrations across quartiles of glutathione-related oxidative potential (OP^{GSH})

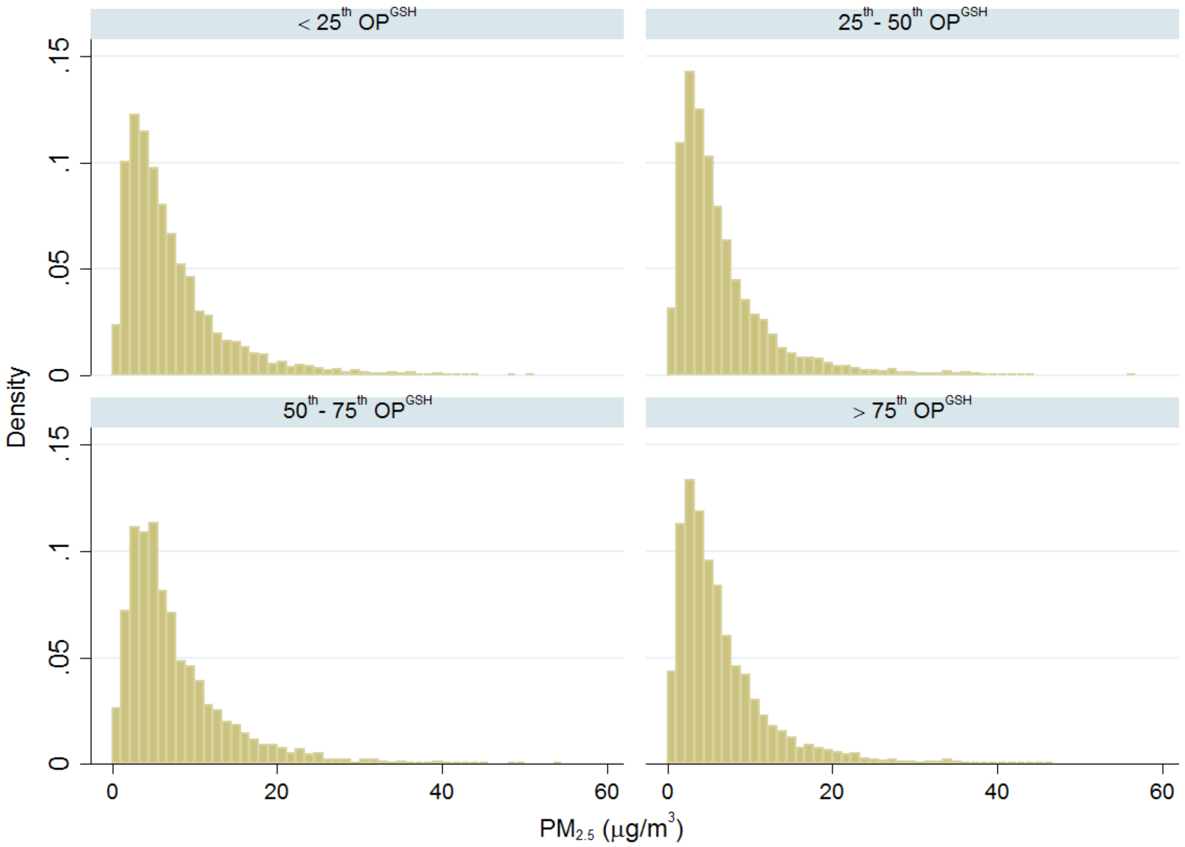

Figure S4. Ambient $PM_{2.5}$, $PM_{2.5}$ oxidative burden and emergency room visits for myocardial infarction: sensitivity analyses excluding sites with less than 1-month of oxidative potential data. Risk estimates reflect a 5 $\mu g/m^3$ increase in lag-0 $PM_{2.5}$ and 1 unit changes in $PM_{2.5}$*GSH and $PM_{2.5}$*AA. All models are adjusted for 3-day mean ambient temperature and relative humidity (cubic splines).

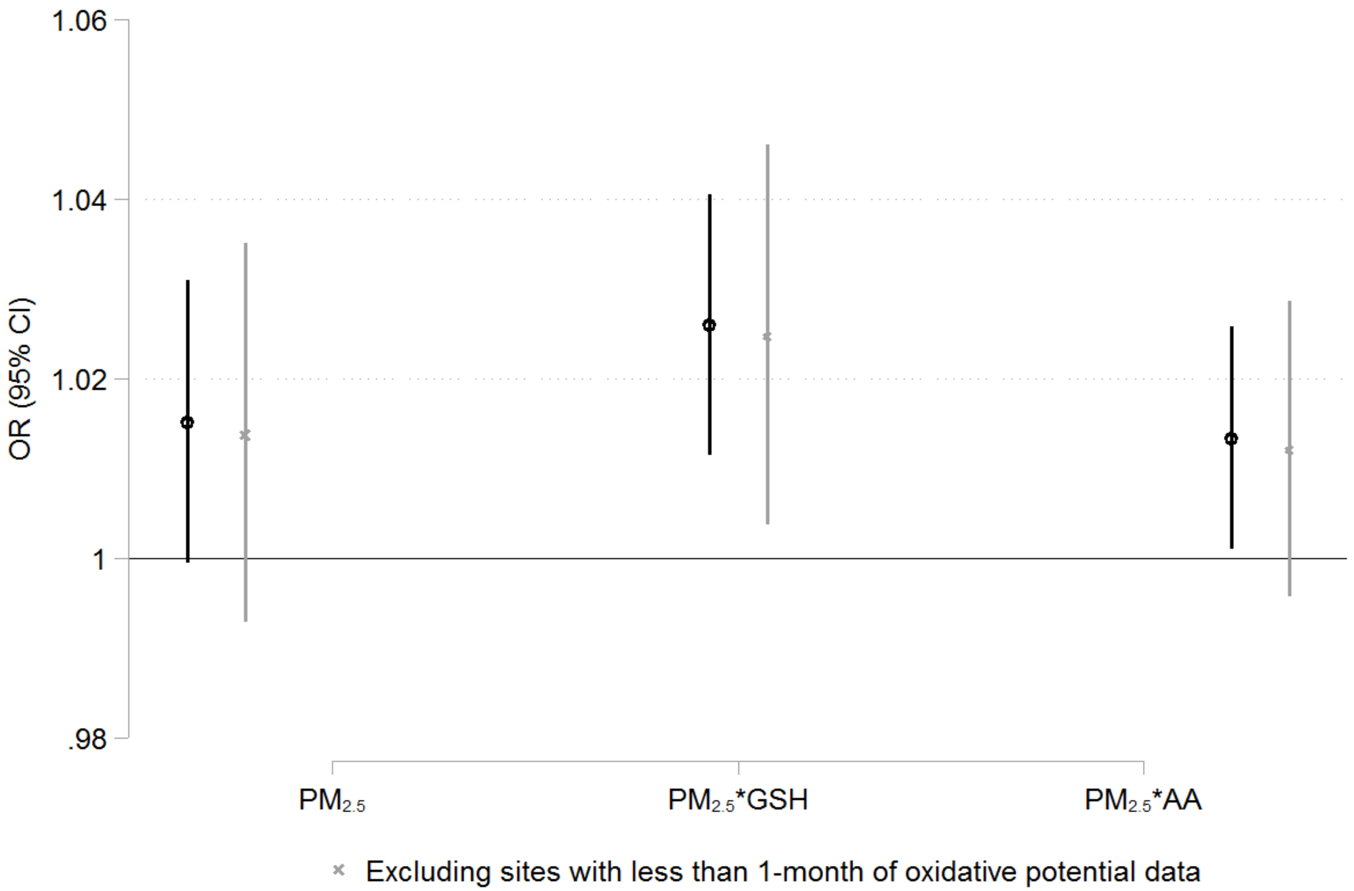

Figure S5. Concentration response plots (using cubic splines with 4 knots) for NO_2, O_3, and O_x and risk of emergency room visits for myocardial infarction adjusted for lag-0 $PM_{2.5}$*GSH and 3-day mean ambient temperature and relative humidity. Slopes for NO_2 and O_3 increase in two-pollutant models (i.e. including NO_2 and O_3) compared to single pollutant models.

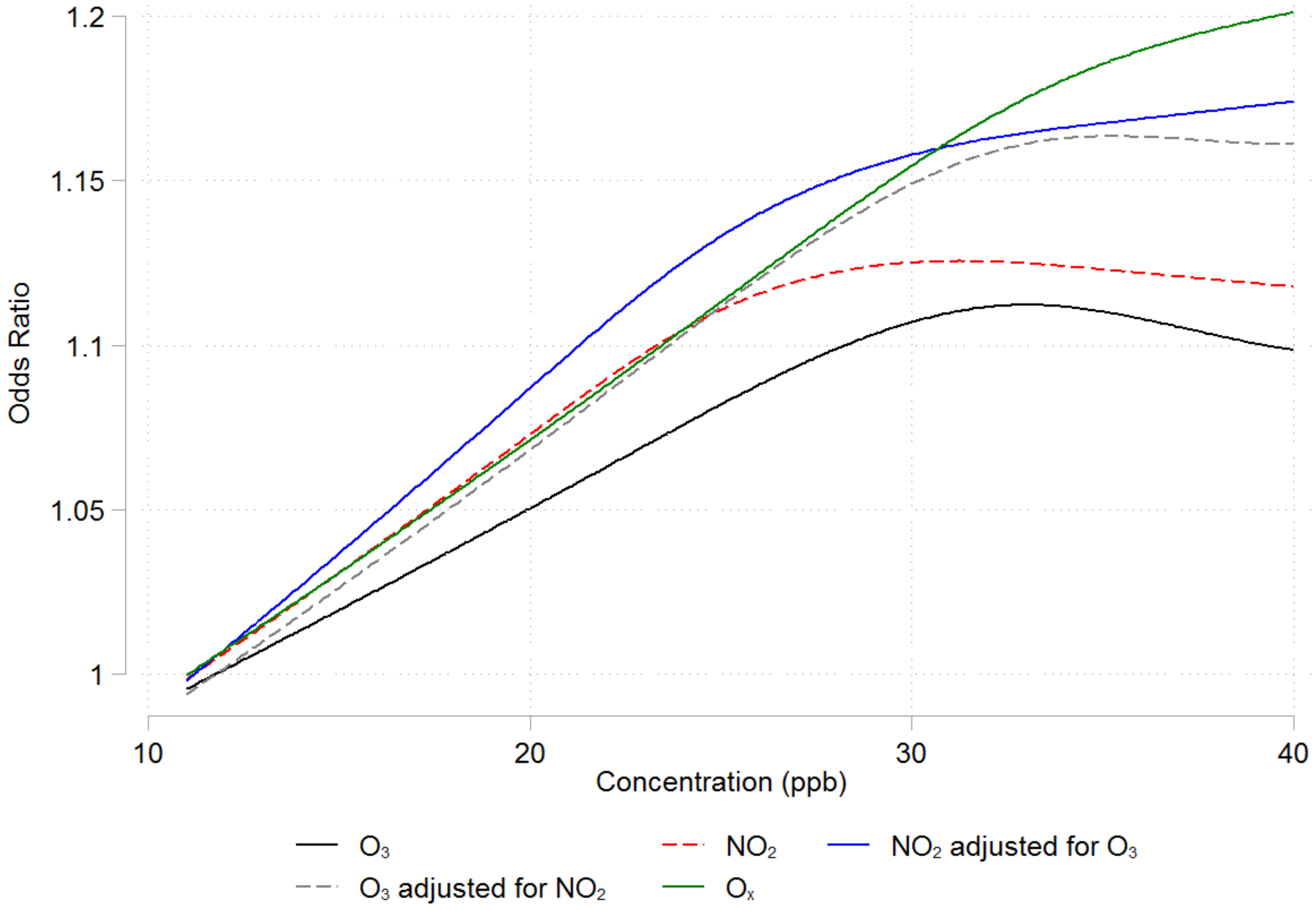

Figure S6. Concentration response plots for $PM_{2.5}$ and risk of emergency room visits for myocardial infarction above the 90th percentile of OP^{GSH} (black line) and below the 50th percentile (gray line) using cubic splines with 4 knots. All models are adjusted for 3-day mean ambient temperature and relative humidity.

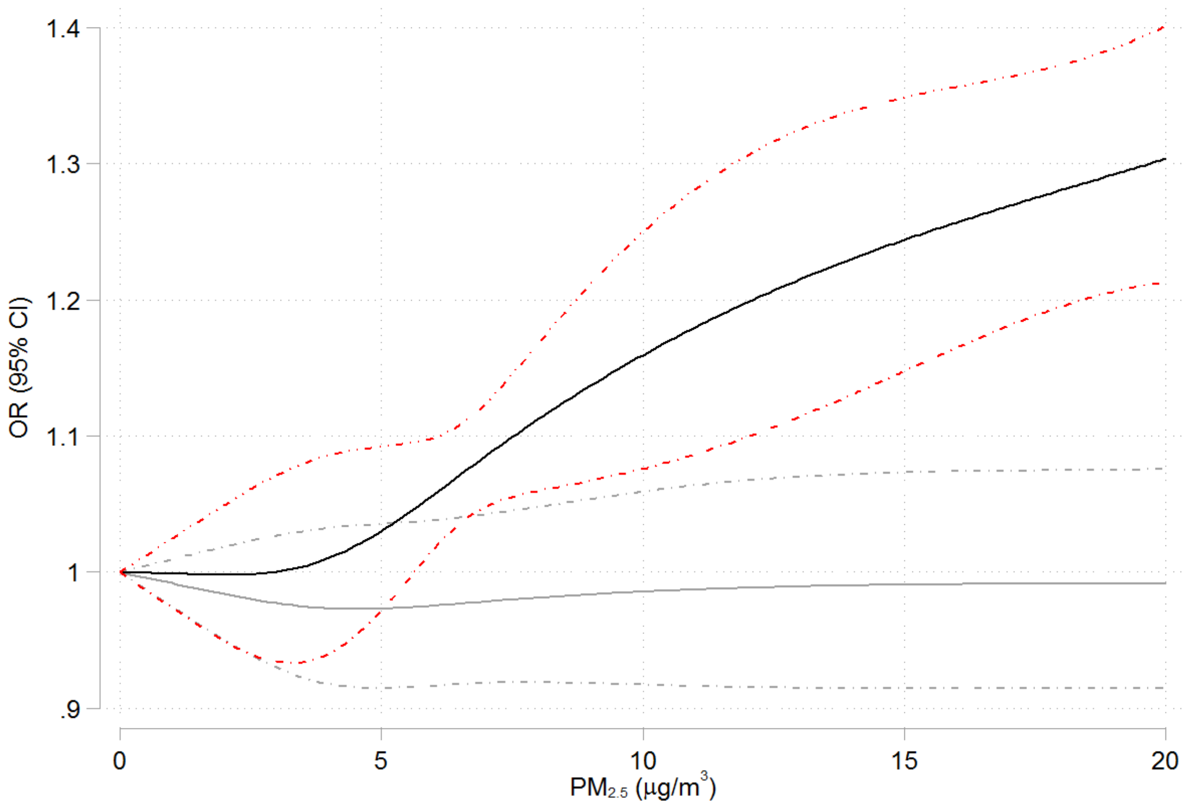

Supplement: Additional file 3: — (Figure S1: Total sampling days for regional oxidative potential (OP) at each site (2012–2013). Values below the 10th percentile (30 days, indicated by the red vertical line) were excluded for sensitivity analyses; Figure S2: Regional estimates of glutathione (OPGSH) and ascorbate (OPAA)-related oxidative potential in Ontario, Canada (2012–2013); Figure S3: Distribution of daily PM2.5 mass concentrations across quartiles of glutathione-related oxidative potential (OPGSH); Figure S4: Ambient PM2.5, PM2.5 oxidative burden and emergency room visits for myocardial infarction: sensitivity analyses excluding sites with less than 1-month of oxidative potential data; Figure S5: Concentration response plots (using cubic splines with 4 knots) for NO2, O3, and Ox and risk of emergency room visits for myocardial infarction; Figure S6: Concentration response plots for PM2.5 and risk of emergency room visits for myocardial infarction above the 90th percentile of OPGSH and below the 50th percentile using cubic splines with 4 knots). (PDF 382 kb) [file 12940_2016_129_MOESM3_ESM.pdf]
